# Supplementary material for: Hemolysin Co-Regulatory Protein 1 Enhances the Virulence of Clinically Isolated Escherichia coli in KM Mice by Increasing Inflammation and Inducing Pyroptosis
Source: Toxins (Basel). 2023 Feb 22;15(3):171. doi: 10.3390/toxins15030171 (PMC10058142; doi:10.3390/toxins15030171)
Supplement: Supplementary file 1 [file toxins-15-00171-s001.zip › toxins-2146755-supplementary.pdf]

toxins

Article

Hemolysin Co-Regulatory Protein 1 Enhances the Virulence of Clinically Isolated *Escherichia coli* in KM Mice by Increasing Inflammation and Inducing Pyroptosis

Hao Wang, Long-bao Lv, Li-ping Chen, Jin-long Xiao, Jue Shen, Bin Gao, Jin-gang Zhao, Dong-mei Han, Bin-xun Chen, Shuai Wang, Gen Liu, Ai-guo Xin, Peng Xiao and Hong Gao

A ttgacagctagctcagtcctaggtataatactagtNNNNNNNNNNNNNNNNNNNNGTTTTAGAGCTAGAAAT
AGCAAGTTAAAATAAGGCTAGTCCGTTATCAACTTGAAAAAGTGGCACCGAGTCGGTG
CTTTTTTT

B

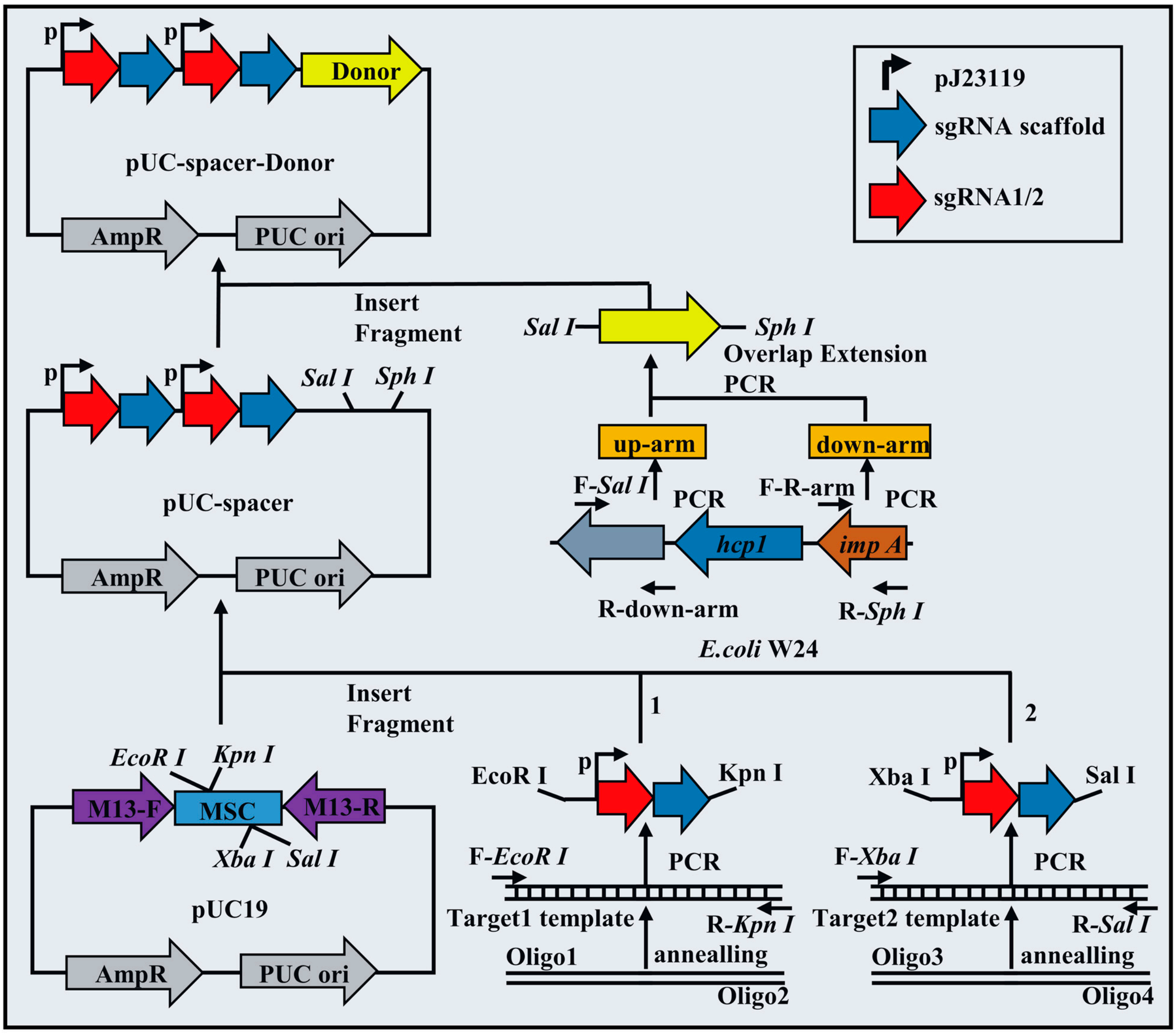

Figure S1. sgRNA expression cassette and plasmid construction process. **(A)**. sgRNA expression cassette. **(B).** Plasmid construction process.

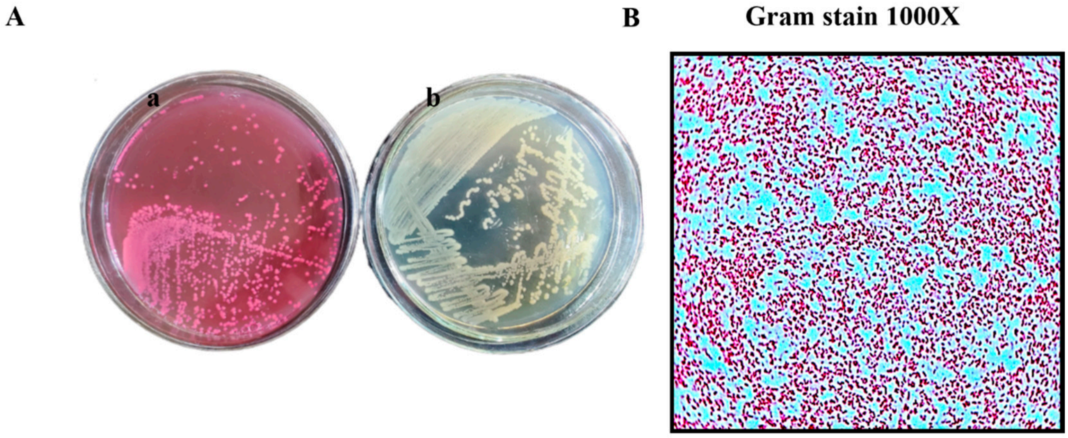

Figure S2. Clinical isolation of *E. coli*. **(A)**. a. *E. coli* is grown on MacConkey medium, b. *E. coli* is grown on LB medium. **(B)**. Gram staining of *E. coli*.

Table S1. Strains and plasmids used in this study.

Strain or plasmid	Characteristic(s)	Source
Strains		
W24	A clinical isolate from the faeces of a piglet with diarrhea; EPEC	aboratoy stoc
DH5α	F^- ,φ80d*lacZ* ΔM15, Δ(*lacZYA-argF*)U169, *deoR* , *recA1* , *endA1* , *hsdR17* (r_K^- , m_K^-), *phoA*, *supE*44 , λ^- , *thi*-1, *gyrA*96 , *relA*1	TaKaRa
W24Δ*hcp1*	W24 mutant with hcp1 deletion	This study
Plasmids		
pUC19	Amp^r, pMBI origin ,DNA sequencing using M13 series primers	TaKaRa
pCas	Kan^r,ParaBAD-λ-Red recombinase, Pcas-cas9,Ptrc-pMBI-sgRNA,pSC101ts origin	aboratoy stoc
pUC-spacer-Donor	Based on pUC19 skeleton,sgRNA1-Hcp1, sgRNA2-Hcp1,Donor	This study

Table S2. Primers used for the PCR amplification.

Primers	Primer sequences
up-arm-F-*Sal I*	***ACGCGTCGAC***AACTAACCAGGCGAGTGTAT
up-arm -R	GCCAGAGGCGGCAACCGGTACTTACTGTAATAACATAATA
down-arm-F	TATTATGTTATTACAGTAAGTACCGGTTGCCGCCTCTGGC
down-arm-R *Sph I*	***ACATGCATGC***CAGCAACAGGCCTCGACACAATG
Oligo1-F	tcctaggtataatactagt**GAATGCAATGAGTAAATGGC**GTTTTAGAGCTAGAAATAGCAAGTTAAAATA GGCTAGTCCGT
Oligo1-R	ACGGACTAGCCTTATTTTAACTTGCTATTTCTAGCTCTAAAAC**GCCATTTACTCATTGCATTC**acta tattatacctagga
Oligo2-F	tcctaggtataatactagt**ATAAAGGCAATTCTCTCCTG**GTTTTAGAGCTAGAAATAGCAAGTTAAAATAA GGCTAGTCCGT
Oligo2-R	ACGGACTAGCCTTATTTTAACTTGCTATTTCTAGCTCTAAAAC**CAGGAGAGAATTGCCTTTAT**act gtattatacctagga
F-*EcoR I*	***CCGGAATTC***ttgacagctagctcagtcctaggtataatactagt
R-*Kpn I*	***CGGGGTACC***AAAAAAAGCACCGACTCGGTGCCACTTTTTCAAGTTGATAACGGACTAGCCTTATT TA
F- *Xba I*	***CTAGTCTAGA***ttcttgacagctagctcagtcctaggtataatactagt

R- *Sal I*	***ACGCGTCGAC***AAAAAAAGCACCGACTCGGTGCCACTTTTTCAAGTTGATAACGGACTAGCCTTAT TTTA
pCas-JD-F	TCCGCCTGTCTGTACTTCTG
pCas-JD-R	CGTCGTTGGAACTGCTTTGA
M13-F	GTAAAACGACGGCCAGT
M13-R	CAGGAAACAGCTATGAC
QSJD-F	CAATAGCGAGTTCCAGAACT
QSJD-R	AGATGATGCGTGTTGCCGAC

Table S3. Primers used for qPCR analysis.

Name	Sequences
β-actin	CACCACACCTTCTACAATGA
**NM_007393.5**	GTCTCAAACATGATCTGGGT
NLRP3	GAGTCTAGCAGACCTGATTG
**KF032621.1**	TTGTAGCTCATCAAAGCCAT
ASC	GCTCTTGAAAACTTGTCAGG
**AB059327.1**	CATGTCTCTAAGCACAGTCA
Caspase-1	GTACACGTCTTGCCCTCATT
**NM_009807.2**	TCACGGTATACCCCAGATCC
IL-1β	GTGATATTCTCCATGAGCTTTGTAC
**NM_008361.4**	CATTACACAGGACAGGTATAGATTC
IL-18	TGTCTACCCTCTCCTGTAAG
**NM_008360.2**	AAGCAAGAAAGTGTCCTTCA
GSDMD	TGACAGTTCCAGTGCCT
**NM_026960.4**	CCTCGGTCACCACAAAC

Table S4. Docking scores and confidence scores.

**Rank**	**1**	**2**	**3**	**4**	**5**	**6**	**7**	**8**	**9**	**10**
**Docking Score**	-266.70	-264.71	-260.41	-258.40	-253.89	-253.57	-230.70	-228.65	-224.58	-223.83
**Confidence Score**	0.9117	0.9084	0.9010	0.8973	0.8887	0.8881	0.8340	0.8282	0.8163	0.8141
**Ligand rmsd (Å)**	87.10	107.29	92.66	102.68	102.67	93.32	119.23	121.56	94.18	137.16

Table S5. Reaction system and procedure for qPCR analysis.

constituents	reaction system	temperature	time	
SYBR Premix Ex Taq Ⅱ	10.0 μl	37℃	10 min	10 cycles
Forward/Reverse Primer（10μM）	0.8 μl	16℃	10 min	
Template cDNA	2.0 μl	50℃	5 min	
dH2O	6.4 μl	65℃	20 min	
Total	up to 20 μL	4℃	∞	
